# Cerebral vasculitis and lateral rectus palsy – two rare central nervous system complications of dengue fever: two case reports and review of the literature

**DOI:** 10.1186/s13256-018-1627-x

**Published:** 2018-04-19

**Authors:** H. M. M. Herath, J. S. Hewavithana, C. M. De Silva, O. A. R. Kularathna, N. P. Weerasinghe

**Affiliations:** 1 Department of Medicine, Faculty of Medicine, University of Ruhuna , P.O. Box 70, Galle, Sri Lanka; 2 University Unit, Karapitiya Teaching Hospital, Galle, Sri Lanka; 3 Department of Microbiology, Faculty of Medicine, University of Ruhuna, Galle, Sri Lanka

**Keywords:** Dengue infection, Cerebral vasculitis, Encephalitis, Cranial nerve palsy, Neurological complications

## Abstract

**Background:**

Dengue fever is a common mosquito-borne viral illness with a clinical spectrum ranging from a simple febrile illness to potentially life-threatening complications such as dengue hemorrhagic fever and dengue shock syndrome. Dengue infection can affect many organs, including the central nervous system. The neurological manifestations reported in dengue infections are meningitis, encephalitis, stroke, acute disseminated encephalomyelitis, and Guillain-Barré syndrome.

**Case presentation:**

We report the cases of two interesting patients with confirmed dengue infection who presented with complications of possible central nervous system vasculitis and cranial nerve palsy. The first patient was a 53-year-old previously healthy Singhalese woman who developed acute-onset slurring of speech and ataxia with altered sensorium 1 day after recovery from a critical period of dengue hemorrhagic fever. Subsequent investigations revealed evidence of encephalopathy with brainstem ischemic infarctions. Her clinical picture was compatible with central nervous system vasculitis. She was treated successfully with intravenous steroids and had a full functional recovery. The second patient was a middle-aged Singhalese woman who had otherwise uncomplicated dengue infection. She developed binocular diplopia on day 4 of fever. An ocular examination revealed a convergent squint in the left eye with lateral rectus palsy but no other neurological manifestation.

**Conclusions:**

Central nervous system vasculitis due to dengue infection is a very rare phenomenon, and to the best of our knowledge, only one case of central nervous system vasculitis has been reported to date, in a patient of pediatric age. Cranial nerve palsy related to dengue infection is also rare, and only a few cases of isolated abducens nerve palsy have been reported to date. The two cases described in this report illustrate the rare but important central nervous system manifestations of dengue fever and support the fact that the central nervous system is one of the important systems that can be affected in patients with dengue infection.

## Background

Dengue fever is a common arboviral infection in tropical and subtropical areas of the world. It is transmitted by the hematophagous mosquito *Aedes aegypti* [1]. Around 2.5 billion people in more than 100 countries are at risk of dengue infection, with about 100 million cases of symptomatic dengue occurring annually [2]. Being a tropical country with high rainfall, Sri Lanka has experienced frequent dengue outbreaks for the last several decades. In 2017, over 100,000 cases of dengue infection were reported in Sri Lanka [3]. Dengue infection can be caused by one of four distinct, antigenically related serotypes: DEN-1, DEN-2, DEN-3, and DEN-4 [1].

The clinical presentation of dengue infection ranges from mild clinical febrile illness to potentially life-threatening dengue hemorrhagic fever (DHF) and dengue shock syndrome (DSS) [4]. The main cause of death in dengue infection is either DHF or DSS, both of which are due to increased capillary permeability resulting in plasma leakage [5]. Apart from these complications, dengue infection is known to cause multiple organ involvement, including the central nervous system (CNS) [6–10]. Neurological complications in dengue infection can be explained by three different pathogenic mechanisms: (1) direct invasion of the CNS, leading to encephalitis, meningitis and myelitis; (2) systemic complications resulting in encephalopathy and stroke; and (3) para- or postinfectious immune-mediated effects, such as acute disseminated encephalomyelitis (ADEM), Guillain-Barré syndrome, and optic neuritis [7, 11].

We report two cases of patients with dengue infection associated with CNS complications. The first patient had possible CNS vasculitis related to dengue, and the second had isolated cranial nerve (abducens nerve) palsy. These two cases are of importance because, to the best of our knowledge, CNS vasculitis triggered by dengue infection has not been described previously in the literature. Furthermore, only three cases of isolated cranial nerve palsy related to dengue infection have been reported previously.

## Case presentation

### Patient 1

An otherwise healthy 53-year-old Singhalese woman who was an office worker from Southern Province, Sri Lanka, presented to a local hospital with an acute febrile illness with no apparent focus of infection. She had no significant past medical illnesses and did not smoke or consume alcohol. Three days prior to her hospital admission, she developed high fever with chills, myalgia, and moderate headache. She was conscious and rational with no hemodynamic compromise. Her full blood count revealed a hemoglobin level of 13.6 g/dl, packed cell volume (PCV) of 37.9%, white blood cell count of 4.2 × 10^3^/mm^3^, and platelet count of 105 × 10^3^/mm^3^. The result of her nonstructural protein 1 (NS1) antigen test (enzyme-linked immunosorbent assay) was positive. Her liver transaminases were elevated with aspartate aminotransferase (AST) of 161 U/L and alanine aminotransferase (ALT) of 113 U/L. In the next 48 hours, she was observed in the local hospital with serial full blood count measurements, which showed a gradual reduction of her platelet count to 41 × 10^3^/mm^3^ on the fifth day of her illness, at which point her PCV had risen to 42%. She had evidence of free fluid in the hepatorenal pouch on an ultrasound scan, indicating progression into the critical phase of DHF. She was observed and managed in the next 48 hours of the critical phase according to the established national guidelines on dengue management [12]. She went through the critical phase without any significant complication, such as bleeding, shock, or multiorgan failure. Following resolution of the critical phase, she was afebrile and clinically improving. Her PCV dropped from 42% to 36.5%, and her platelets showed a gradual increase to 141 × 10^3^/mm^3^. However, on postcritical day 1, she experienced acute-onset slurring of speech, deviation of the mouth, difficulty in swallowing, and unsteadiness in gait. At this juncture, she had no fever, headache, or seizures. An urgent noncontrast computed tomographic scan did not demonstrate any particular abnormality in the brain and brainstem. The patient was transferred to our unit for further evaluation and management.

**Fig. 1:**
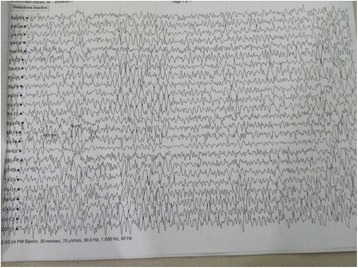
Electroencephalogram showing generalized spike and wave discharges and periodicity compatible with encephalopathy

**Fig. 2:**
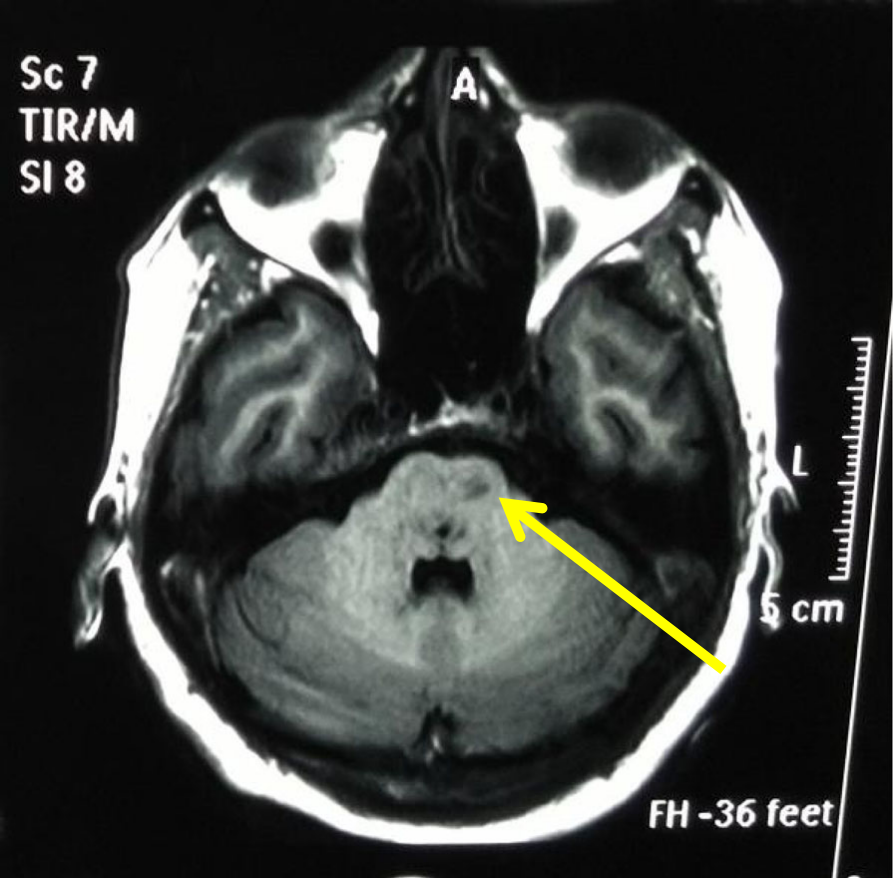
Magnetic resonance imaging scan of the brainstem showing multiple infarctions involving the pons and medulla regions (*arrow*)

On admission to our unit, the patient was hemodynamically stable with a blood pressure of 112/74 mmHg and pulse rate of 84 beats/minute. She was afebrile but was slightly drowsy with a Glasgow Coma Scale score of 13/15. She had right-sided lower motor-type facial nerve (cranial nerve VII) palsy with ipsilateral palatal weakness, horizontal nystagmus with fast component to the right, and mild weakness in the left upper and lower limbs with normal reflexes and sensory and plantar responses. The rest of the examination, including funduscopy, was unremarkable. Subsequent laboratory investigations, including septic screening with blood culture and mycoplasma serology, serum electrolytes, and liver and renal function, did not reveal any significant abnormalities. Apart from leukopenia (total white cell count of 3400/mm^3^, 42% neutrophils, 56% lymphocytes), a repeat full blood count showed no significant abnormalities. The diagnosis of dengue infection was reconfirmed by the presence of dengue-specific immunoglobulin G (IgG) and IgM antibodies in serum on day 7 of the patient’s illness (ELISA method). Analysis of the patient’s cerebrospinal fluid (CSF) revealed a normal protein level of 53 g/L, a normal glucose level, and no pleocytosis. An electroencephalogram (EEG) showed features of encephalitis (Fig. 1). Magnetic resonance imaging (MRI) of the brain revealed multiple infarctions involving the pons and medulla regions (Fig. 2).

The patient was treated with intravenous dexamethasone to reduce cerebral inflammation. Supportive measures, including physiotherapy and speech therapy, were initiated. She had gradual but progressive improvement over the next few weeks and had a full functional recovery around 1 month after the illness. CNS vasculitis due to dengue infection was the presumptive diagnosis, based on the clinical presentation of acute onset of focal neurological signs with evidence of brainstem infarction and encephalopathy.

### Patient 2

A 57-year-old previously healthy Singhalese woman who was a schoolteacher was admitted to our unit with a history of fever, headache, malaise, nausea, and anorexia of 3 days’ duration. She did not smoke or use alcohol or recreational drugs. On examination, she had a temperature of 39.5 °C and was hemodynamically stable with a pulse rate of 66 beats/minute and blood pressure of 112/66 mmHg with no significant postural drop. The rest of her examination was unremarkable. Her full blood count revealed a total leukocyte count of 4100/mm^3^ with 42% lymphocytes, hemoglobin of 13.4 g/dl, PCV of 40%, platelet count of 94,000/mm^3^, and elevated liver transaminases (ALT of 77 U/L and AST of 86 U/L). Dengue infection was confirmed by detection of the NS1 antigen.

The patient was started on treatment for uncomplicated dengue fever. However, on the second day of her hospital admission, she started complaining of bilateral diplopia, which worsened upon left gaze. She did not complain of headache, and her consciousness level was not reduced at this stage. An ocular examination revealed a convergent squint in the left eye with evidence of isolated lateral rectus weakness. The results of the rest of the CNS examination, including the vision and fundus of both eyes, were normal. The patient was investigated with an MRI scan of the brain, which ruled out a space-occupying lesion or any other significant pathology. During the patient’s hospital stay, her platelet count dropped gradually to 44,000/mm^3^ on the fifth day of fever, before it started to rise. Apart from isolated lateral rectus weakness, she did not develop any other complications, such as plasma leakage, shock, hemorrhage, or liver failure.

She was managed with intravenous and oral fluids according to the national guideline for the management of dengue fever [12]. She recovered from dengue fever and was discharged from the hospital on day 7 of her illness. She had evidence of improvement of her lateral rectus palsy at the time of discharge, and it completely resolved within 4 weeks without any specific treatment.

## Discussion

The two patients with dengue infection whom we describe in this report, one with possible CNS vasculitis and the other with isolated cranial nerve (abducens nerve) palsy, had extremely rare CNS manifestations of dengue infection. Particularly, CNS vasculitis triggered by dengue infection has not been described in the literature previously, to the best of our knowledge. Furthermore, only three cases of isolated cranial nerve palsy related to dengue infection have been reported previously.

Dengue infections pose a significant health challenge in affected countries in the tropics [2]. CNS manifestations are increasingly recognized and can cause diagnostic and management dilemmas [13]. A wide variety of CNS manifestations of dengue have been described, ranging from meningitis [14–16] to immune-mediated disease such as Guillain-Barré syndrome [17, 18] and optic neuritis [19]. CNS involvement of dengue infection was first described by Sanguansermsri *et al*. in 1976 in a patient who presented with encephalopathy [20]. Since then, many different types of CNS involvement have been described. Even though the pathogenesis of CNS involvement of dengue infection is still poorly understood, direct viral invasion, autoimmune reaction, and metabolic and hemorrhagic disturbances are widely considered as possible mechanisms. Murthy categorized CNS manifestations in dengue infection into three groups [21]: (1) CNS involvement due to direct neurotropic effects such as meningitis, encephalitis, and myelitis; (2) CNS involvement due to metabolic complications such as encephalopathy, hypokalemic paralysis, and stroke; and (3) postviral immune-mediated CNS complications such as encephalomyelitis, optic neuritis, and Guillain-Barré syndrome [21].

Based on patient 1’s presentation and investigations, CNS vasculitis was considered as the most likely diagnosis. Most patients with isolated CNS vasculitis typically have headache and features of encephalopathy, with evidence of multiple cerebral infarctions [22]. Patient 1 developed ischemic stroke, as evidenced by acute onset of focal neurological symptoms (dysarthria and ataxia) and signs of brainstem lesions (such as palatal palsy and lower motor facial nerve palsy) with evidence of brainstem infarctions involving the pons and medulla regions on an MRI scan. In addition, patient 1 had clear evidence of encephalopathy, as indicated by altered sensorium and typical EEG abnormalities observed in encephalopathy. Alternative causes for patient 1’s encephalopathy were considered unlikely, given that there was no evidence of hemodynamic compromise, metabolic derangement such as hypoglycemia, electrolyte imbalance, or liver failure. This was further confirmed by the presence of a normal CSF study. Dengue encephalitis was considered as one of the possibilities. However, the combination of stroke with multiple infarctions, encephalopathy with altered sensorium, headache, and absence of CNS pleocytosis made CNS vasculitis more likely. Furthermore, the main features of dengue encephalitis, such as seizures and CNS pleocytosis, were absent in patient 1 [23]. Even though CNS pleocytosis may be absent in some patients with dengue encephalitis, it is a well-recognized feature of the disease [13, 15, 23]. For example, the diagnostic criteria for dengue encephalitis proposed by Carod-Artal *et al*. included CSF pleocytosis [11].

There are many challenges in making a definitive diagnosis of isolated cerebral vasculitis [22]. The lack of uniform consensus regarding diagnostic criteria, as well as diagnostic limitations of specific investigations such as angiography and imaging, present important challenges. Angiography was not considered in patient 1 because she responded to the treatment well and she was unwilling to undergo further testing due to her prolonged hospital stay. CNS vasculitis due to dengue infection is a very rare phenomenon, and to the best of our knowledge, only one case of CNS vasculitis has been reported to date, in a patient of pediatric age [24]. As far as we are aware, there are no previous cases of CNS vasculitis reported in adults with dengue.

Regarding treatment, the mainstay of treatment for dengue infection and its associated CNS complications is supportive therapy based on locally available guidelines [7, 12, 23, 25]; however, steroids may have a place in treating some of the immune-mediated CNS complications. There is some evidence that CNS complications such as ADEM [26, 27] and myelitis [25, 28] have a favorable response to steroid therapy. Patient 1 also had a good response to an intravenous steroid (dexamethasone), which may suggest that an underlying immune-mediated pathological process was the most likely reason for the CNS manifestation.

Patient 2 had isolated sixth cranial nerve palsy that developed on the fourth day of her illness. Cranial nerve palsy associated with dengue infection is also relatively rare, with only a few cases reported previously [8, 29]. Optic neuropathy [19], oculomotor nerve palsy [30], and facial nerve palsy [9, 31] associated with dengue infection have been described previously. Abducens nerve palsy has also been described, but only in a few cases [8, 9, 29]. All reported cases of abducens nerve palsy were in male patients, and none of them had features of DHF (plasma leakage) or DSS. Most patients developed abducens nerve palsy roughly 1 week after the onset of fever. However, patient 2 and the patient reported by Mazliha *et al*. [9] developed abducens nerve palsy within the first 5 days of the onset of fever (Table 1). Even though some authors have suggested that the underlying pathophysiology behind cranial palsy could be due to an immune-mediated mechanism, direct viral invasion of cranial nerves is more likely, considering the fact that some patients developed abducens nerve palsy at an early stage of their illness (days 2–4). The fact that all patients recovered fairly rapidly without any specific therapies such as steroids favors direct viral invasion over immune-mediated mechanisms [7, 9].

**Table 1:** Characteristics of previously reported patients with abducens nerve palsy

Study	Patient age (years)	Sex	Day of onset of mononeuropathy in relation to fever	Type of mononeuropathy	Associated with DHF or DSS	Associated with other CNS manifestation
Mishra, *et al*. [29]	52	Male	Day 8	Lateral rectus palsy	No	No
Shivanthan MC *et al*.	29	Male	Day 7	Lateral rectus palsy	No	No
Mazliha, *et al*. [9]	40	Male	Day 2	Lateral rectus	No	No

*Abbreviations: CNS* Central nervous system, *DHF* Dengue hemorrhagic fever, *DSS* Dengue shock syndrome

Specific therapies, such as steroids or immunoglobulins, are not generally required in the management of dengue-related abducens nerve palsy [8, 9, 29]. Patient 2 was managed with supportive therapy with fluid and paracetamol during the acute stage. She had a complete recovery from the nerve palsy within the first 4 weeks of the illness. Similar to patient 2, other patients with dengue-associated cranial nerve palsies, including abducens nerve palsy, Bell’s palsy, and oculomotor nerve palsy, have been reported to improve without any specific therapy [8, 9]. The prognosis for dengue-related cranial nerve palsies is good, and complete recovery was observed in all previously reported cases [8, 9, 25].

## Conclusions

Even though most cases of dengue infection have a self-limiting course, some patients develop devastating and life-threatening complications such as DHF and DSS. In addition to these two complications, dengue infection can affect many other organ systems, including the CNS. Even though a wide variety of CNS manifestations of dengue infection have been reported, CNS vasculitis associated with dengue infection has not been described previously among adults. To the best of our knowledge, we report the first case of CNS vasculitis in an adult patient with dengue infection. Furthermore, abducens nerve palsy in the setting of fever in a tropical country such as Sri Lanka could very well be due to dengue infection, and dengue infection should be considered as a cause of abducens nerve palsy.

## Abbreviations

ADEM: Acute disseminated encephalomyelitis
ALT: Alanine aminotransferase
AST: Aspartate aminotransferase
CNS: Central nervous system
CSF: Cerebrospinal fluid
DHF: Dengue hemorrhagic fever
DSS: Dengue shock syndrome
EEG: Electroencephalogram
ELISA: Enzyme-linked immunosorbent assay
Ig: Immunoglobulin
MRI: Magnetic resonance imaging
NS1: Nonstructural protein 1
PCV: Packed cell volume
